# Spinal Muscular Atrophy: From Defective Chaperoning of snRNP Assembly to Neuromuscular Dysfunction

**DOI:** 10.3389/fmolb.2017.00041

**Published:** 2017-06-08

**Authors:** Maia Lanfranco, Neville Vassallo, Ruben J. Cauchi

**Affiliations:** ^1^Department of Physiology and Biochemistry, Faculty of Medicine and Surgery, University of MaltaMsida, Malta; ^2^Center for Molecular Medicine and Biobanking, University of MaltaMsida, Malta; ^3^Institut de Génétique Moléculaire de Montpellier, Center National de la Recherche Scientifique-UMR 5535, Université de MontpellierMontpellier, France

**Keywords:** survival motor neuron, SMN-Gemins complex, snRNP assembly, missplicing, motor neuron disease (MND), amyotrophic lateral sclerosis (ALS), spinal muscular atrophy (SMA), spliceosome

## Abstract

Spinal Muscular Atrophy (SMA) is a neuromuscular disorder that results from decreased levels of the survival motor neuron (SMN) protein. SMN is part of a multiprotein complex that also includes Gemins 2–8 and Unrip. The SMN-Gemins complex cooperates with the protein arginine methyltransferase 5 (PRMT5) complex, whose constituents include WD45, PRMT5 and pICln. Both complexes function as molecular chaperones, interacting with and assisting in the assembly of an Sm protein core onto small nuclear RNAs (snRNAs) to generate small nuclear ribonucleoproteins (snRNPs), which are the operating components of the spliceosome. Molecular and structural studies have refined our knowledge of the key events taking place within the crowded environment of cells and the numerous precautions undertaken to ensure the faithful assembly of snRNPs. Nonetheless, it remains unclear whether a loss of chaperoning in snRNP assembly, considered as a “housekeeping” activity, is responsible for the selective neuromuscular phenotype in SMA. This review thus shines light on *in vivo* studies that point toward disturbances in snRNP assembly and the consequential transcriptome abnormalities as the primary drivers of the progressive neuromuscular degeneration underpinning the disease. Disruption of U1 snRNP or snRNP assembly factors other than SMN induces phenotypes that mirror aspects of SMN deficiency, and splicing defects, described in numerous SMA models, can lead to a DNA damage and stress response that compromises the survival of the motor system. Restoring the correct chaperoning of snRNP assembly is therefore predicted to enhance the benefit of SMA therapeutic modalities based on augmenting SMN expression.

## Introduction

Spinal Muscular Atrophy (SMA) is a neuromuscular disorder that can afflict both infants and adults. Patients present with loss of lower motor neurons and profound muscle weakness leading to immobility and, in severe cases, respiratory failure and death (Kolb and Kissel, 2011). The recent availability of an effective therapy is the culmination of more than two decades of research aimed at characterizing the molecular genetics underlying the disease following the discovery that SMA is caused by mutation or homozygous deletion of the *survival motor neuron 1* (*SMN1*), a gene encoding the SMN protein. Due to a quirk in human evolution, SMN is also encoded by the highly homologous *SMN2* gene. Nonetheless, a single nucleotide substitution (C/T) in exon 7, converts an exon splicing enhancer to a silencer, hence inducing the omission of exon 7 from most of the *SMN2*-derived mRNA transcripts. This alteration leads to the production of an unstable truncated protein isoform (SMNΔ7) that is rapidly degraded, although in the absence of complete penetrance, full-length, functional SMN is still encoded by a small portion of *SMN2* transcripts that evade exon 7 skipping (reviewed in Burghes and Beattie, 2009). In the context of SMA, the levels of SMN are sufficient to prevent lethality yet not enough to fully compensate for the loss of *SMN1*. The inverse correlation between *SMN2* copy number and SMA severity elevated *SMN2* to the leading disease genetic modifier (Wirth et al., 2013). The antisense oligonucleotide (ASO) nusinersen (marketed as Spinraza), recently approved for a broad patient population, is the first successful output of a campaign aimed at identifying therapeutics that promote exon 7 inclusion in *SMN2* transcripts. The backdrop is a string of favorable results from animal models up to clinical trials, all showing that nusinersen enhanced SMN protein levels adequately enough to improve disease phenotypes (Faravelli et al., 2015; Farrar et al., 2016).

Interestingly, ASOs and other therapeutic approaches that treat SMA by augmenting SMN expression, including chief amongst others viral-mediated SMN gene delivery, and orally bioavailable small molecules that correct *SMN2* splicing, are not the obvious sequel to basic knowledge gained on SMN function. SMN, a ubiquitously expressed protein, is known to partner with Gemins 2–8 and Unrip to form a complex that is indispensable for chaperoning the assembly of small nuclear ribonucleoproteins (snRNPs), core elements of the spliceosome. In this review, we present a refined view of the key snRNP assembly events taking place within the crowded environment of cells, and evaluate the evidence favoring the classification of SMA as a chaperonopathy or a disorder arising from a disturbance in the chaperoning of snRNP assembly with the consequential transcriptome abnormalities as the primary drivers of neuromuscular degeneration in SMA patients. A better understanding of the mechanisms underpinning the disease can open up novel therapeutic routes that complement or accentuate the effect of the mainstream approach.

## Anatomy of the SMN-gemins complex chaperone machine

**Figure 1:**
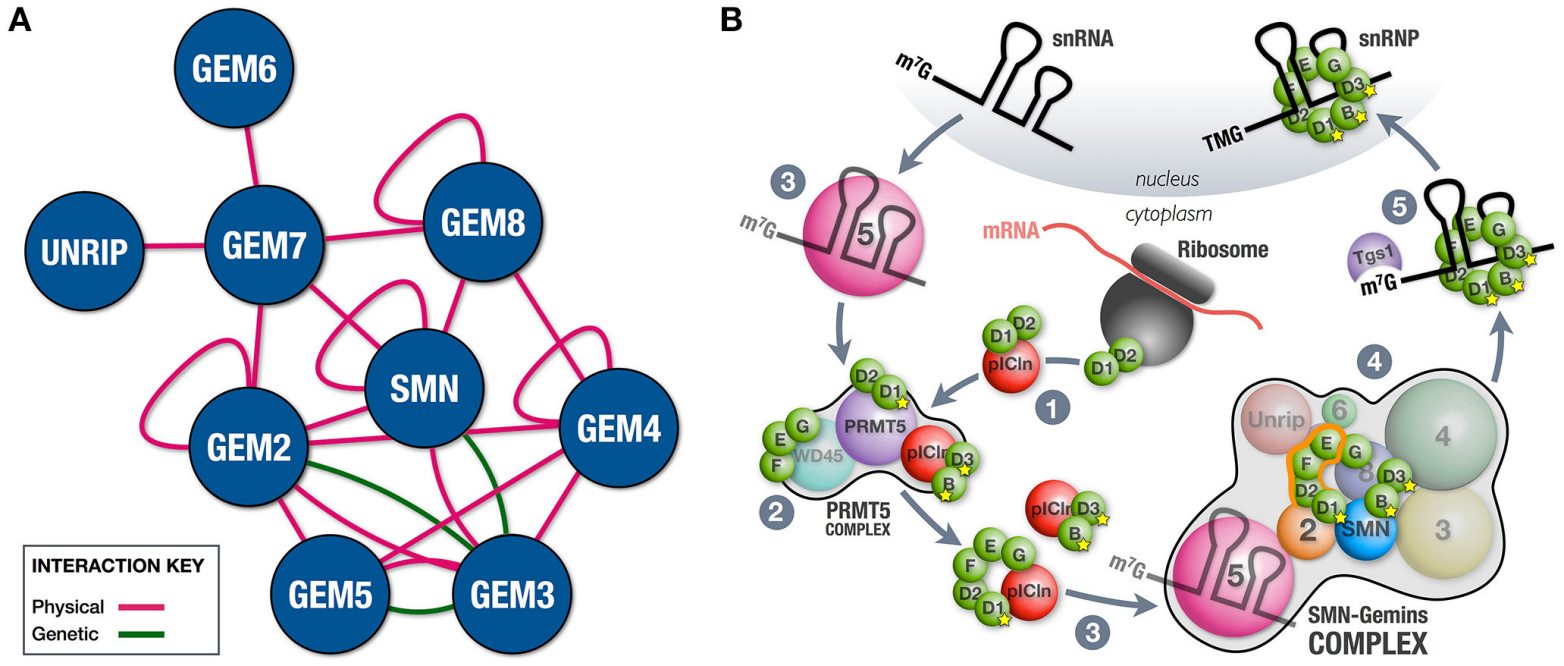
SMN-Gemins complex and its role in chaperoning snRNP assembly. **(A)** Integration of physical and genetic interactions between the constituent members of the SMN-Gemins complex. Physical interactions were identified through various biochemical techniques including two-hybrid, pulldown and co-immunoprecipitation assays in multiple organisms (Lorson et al., 1998; Young et al., 2000; Ogawa et al., 2007, 2009; Otter et al., 2007; Cauchi et al., 2008; Kroiss et al., 2008; Shpargel et al., 2009; Guruharsha et al., 2011). Changes in the levels of SMN or Gemins enhanced viability and motor phenotypes of a hypomorphic Gemin3 mutant in *Drosophila*, thereby allowing the identification of genetic interactions (Borg et al., 2015). Interaction network was composed using the esyN tool. **(B)** Cooperation between the PRMT5 and SMN-Gemins complexes in the cytoplasm ensures the faithful assembly of snRNPs. (1) On translation, Sm D2 protein remains attached to the ribosome. Its release occurs subsequent to the formation of the Sm D2/D1 heterodimer and association with pICln. (2) Select Sm proteins are then post-translationally modified by the PRMT5 complex. (3) The SMN-Gemins complex receives pre-organized Sm subsets and snRNAs from pICln and Gemin5, respectively. (4) Within the SMN-Gemins complex, the majority of Sm proteins are handled by Gemin2 until their uploading onto the delivered snRNAs. (5) Tgs1 ensures cap hypermethylation of assembled snRNPs prior to their nuclear import.

In its simplest version, the SMN-Gemins complex is composed of only SMN (Yab8p) and Gemin2 (Yip1p), a situation that is typical in the fission yeast *Schizosaccharomyces pombe* (Hannus et al., 2000). Complexity was gained in evolution through the incorporation of the remaining constituents (Kroiss et al., 2008; Cauchi, 2010). The fruit fly *Drosophila melanogaster* possesses a minimalistic complex that, in addition to SMN and Gemin2, also includes Gemin3 and Gemin5 (Cauchi et al., 2010). Besides physical associations (Cauchi et al., 2008; Kroiss et al., 2008; Shpargel et al., 2009; Guruharsha et al., 2011), genetic interactions between members of the *Drosophila* SMN-Gemins complex indicate that SMN and its Gemin associates were conserved during evolution not as independent entities but rather as a genetic network (Borg et al., 2015) (Figure 1A). Vertebrates, including humans, have the most elaborate SMN-Gemins complex counting SMN, seven Gemin proteins (Gemin2-Gemin8), and Unrip as its members. Comprehensive biochemical studies revealed a modular composition with the SMN-Gemin8-Gemin7 module placed at its center, thereby allowing the recruitment of the Gemin2-Gemin5 and Gemin6-Unrip subunits mainly via SMN and Gemin7, respectively. The Gemin3-Gemin4 block latches to the complex via both SMN and Gemin8 (Otter et al., 2007). Additional interactions are thought to further stabilize the complex (Otter et al., 2007; Ogawa et al., 2009) (Figure 1A). SMN, Gemin2, Gemin4, and Gemin8 can self-associate (Lorson et al., 1998; Young et al., 2000; Ogawa et al., 2007; Otter et al., 2007), hence their oligomerization propensity means that SMN-Gemins complexes can reach large macromolecular sizes, at least in vertebrates. Cell biology studies have confirmed the clustering of SMN-Gemins complex members to form membrane-less structures named Gems if nuclear (Liu and Dreyfuss, 1996; Cauchi, 2011) or U bodies if cytoplasmic (Liu and Gall, 2007; Cauchi et al., 2010).

It has long been known that loss of SMN is incompatible with life (reviewed in Burghes and Beattie, 2009). The same outcome applies to additional SMN-Gemins complex members investigated thus far. To this end, knockout or RNAi-mediated knockdown of Gemin2, Gemin3 or Gemin5 leads to lethality in various organisms (reviewed in Borg and Cauchi, 2014). This might indicate that the constituents of the SMN-Gemins complex are not redundant, hence, the function of one component cannot be covered by another. Alternatively, or additionally, an imbalance in the protein levels of its members can destabilize the SMN-Gemins complex. Experiments inducing a gain-of-function were revelatory in this regard. Indeed, overexpression of Gemin2 is deleterious in both yeast and flies (Borg et al., 2015). Furthermore, the upregulation of SMN or Gemin5 can have a negative impact on fly viability only when either perturbation is combined with a Gemin3 hypomorphic mutant (Borg et al., 2015). These findings are in line with studies that underscore the interdependence of constituent levels within the SMN-Gemins complex. Hence, cells with low amounts of SMN, including those derived from SMA patients, were found to have reduced protein levels of select Gemins (Jablonka et al., 2002; Helmken et al., 2003; Feng et al., 2005; Shpargel and Matera, 2005; Carissimi et al., 2006; Gabanella et al., 2007; Hao le et al., 2007). In agreement, severe SMA mice were also shown to have a significant reduction in the levels of a subset of Gemin proteins in the spinal cord (Gabanella et al., 2007; Zhang et al., 2008). A similar effect can be achieved on knockdown of select Gemins (Shpargel and Matera, 2005; Ogawa et al., 2007). Furthermore, the half-life of SMN was decreased by mutations interfering with its incorporation within the SMN-Gemins complex (Burnett et al., 2009).

## A refined view of chaperoning activities during snRNP biogenesis

In addition to being an essential step in gene expression, splicing of pre-mRNA transcripts is also crucial for the generation of diverse proteomes in eukaryotes. United in the major spliceosome, U1, U2, U4/U6, and U5 snRNPs catalyze the removal of the majority of pre-mRNA introns. The less abundant minor spliceosome, which processes a rare non-canonical group of introns is however composed of U11, U12, U4atac/U6atac and U5 snRNPs. Not considering the varying number of specific protein components, spliceosomal snRNPs are in essence composed of a short noncoding RNA (snRNA) bound to a heptameric Sm/Lsm protein ring (reviewed in Matera and Wang, 2014). Cells take numerous precautions to ensure the faithful assembly of snRNPs. Hence, key events of the snRNP production cycle take place in the cytoplasm to limit contact of partially assembled snRNPs with their nuclear substrates. Importantly, the process involves cooperation between the SMN-Gemins complex, and the protein arginine methyltransferase 5 (PRMT5) complex, whose constituents include WD45, PRMT5 and pICln. This brings the number of *trans*-acting assembly factors to at least twelve, a count far greater than the parts to be assembled (reviewed in Fischer et al., 2011). The mis-assembly evading measures put forward by cells most probably address the low intrinsic selectively of Sm proteins for snRNAs. As we discuss below, recent molecular and structural studies mostly focusing on Sm-class snRNPs have started to unravel the chaperoning activities of each factor during the uploading of the Sm ring onto a conserved short uridine-rich sequence motif, known as the Sm site, within snRNAs.

snRNP assembly is thought to occur during two phases, the early one dominated by the PRMT5 complex, whereas in the late one, the SMN-Gemins complex is central (Figure 1B). In the early assembly phase, the newly translated Sm D2 protein is thought to remain attached to the ribosome. Formation of the Sm D2/D1 dimer and its association with pICln ensures their release and the subsequent delivery to the PRMT5 complex (Paknia et al., 2016). Here, designated arginine residues of a bound Sm protein subset (B/B', D1, and D3) are symmetrically dimethylated by PRMT5 and, possibly PRMT7, a modification thought to enhance their affinity for the SMN-Gemins complex (reviewed in Fischer et al., 2011). The conclusion of this phase is marked by the formation of the Sm D1/D2/F/E/G and Sm B/D3 sub-complexes, each bound by pICln to prevent premature RNA interactions. Whereas pICln is dismissed, the pre-organized Sm proteins are handed over to the SMN-Gemins complex, a step that signals the initiation of the late assembly phase (Chari et al., 2008; Grimm et al., 2013). Gemin2 is the SMN-Gemins complex subunit that handles the majority of Sm proteins by hugging the crescent-shaped Sm D1/D2/F/E/G pentamer and blocking RNA binding capacity until delivery of snRNAs (Zhang et al., 2011; Grimm et al., 2013). The factor that channels snRNAs to the SMN-Gemins complex is Gemin5 (Battle et al., 2006; Yong et al., 2010), though U1-70K, a component of U1 snRNP, can substitute Gemin5 in a U1-exclusive snRNP assembly pathway (So et al., 2016). Gemin5 is capable of recognizing the Sm site and 7-methylguanosine (m^7^G) cap of nuclear-exported snRNAs via the first and second WD40 repeat domains, respectively (Lau et al., 2009; Jin et al., 2016; Tang et al., 2016; Xu et al., 2016). Although binding to both structures cannot be done simultaneously, this dual recognition tactic is thought to enhance stringency of snRNP assembly. The mechanism ensuring Sm ring closure remains unclear though Unrip and the Gemin6/Gemin7 dimer might have a leading role with the latter thought to act as a temporary substitute for the Sm B/D3 dimer (Ma et al., 2005; Ogawa et al., 2009).

The exact role of other SMN-Gemins complex members in snRNP assembly will probably be unraveled by future mechanistic and structure-based studies. Nevertheless, *in vitro* studies using purified reconstituted systems have recently shown that Gemins 3, 4 and even Gemin5 were dispensable for the assembly and proofreading of snRNAs (Neuenkirchen et al., 2015). This goes against findings by earlier reports demonstrating that snRNP assembly was disrupted on RNAi-mediated knockdown of Gemin3-8 and Unrip in cell culture (Feng et al., 2005; Grimmler et al., 2005; Shpargel and Matera, 2005; Carissimi et al., 2006). It could very well be argued that this outcome is an indirect effect brought about by the destabilization of the SMN-Gemins complex. In support, changes in the levels of its components are known to disrupt the function of the SMN-Gemins complex *in vivo* (Borg et al., 2015). However, it is highly likely that all components of the SMN-Gemins complex have key roles, at least *in vivo*, and their participation in chaperoning snRNP assembly drives the reaction forward in addition to increasing its efficiency. Hence, in an *in vivo* setting, ATP breakdown and RNA or RNP remodeling during snRNP biogenesis is a job most probably attributed to Gemin3, a well-known DEAD-box RNA helicase (Charroux et al., 1999; Yan et al., 2003). In a final step during the late assembly phase, the SMN-Gemins complex recruits trimethylguanosine synthase 1 (Tgs1), an enzyme that hypermethylates the 7-methylguanosine (m^7^G) cap of assembled snRNPs to a 2,2,7-trimethylguanosine (TMG) cap (Mouaikel et al., 2002, 2003). Both the TMG cap and the Sm ring act as a localization signal for their import to the nucleus where they are expected to operate subsequent to a maturation stage in the Cajal body (reviewed in Stanek, 2016).

## *In vivo* studies linking snRNP assembly defects to neuromuscular dysfunction

Several key studies making use of animal models strongly support the possibility that altered snRNP production due to defective chaperoning downstream to SMN deficiency can lead to the neuromuscular defects that are typical in SMA (Table 1). It has long been known that SMN levels strongly stipulate the snRNP assembly capacity of cell extracts (Wan et al., 2005; Boulisfane et al., 2011). In the spinal cord, maximal snRNP assembly chaperoning activity overlaps with the highest demands for SMN during the development of the neuromuscular system (Gabanella et al., 2005; Foust et al., 2010; Le et al., 2011; Lutz et al., 2011; Kariya et al., 2014). Importantly, snRNP assembly capacity in the spinal cord of SMA mouse models was found to determine disease severity, hence, the greatest perturbation was observed in severe SMA mice (Gabanella et al., 2007). In a reciprocal experiment, the disease phenotype was rescued in mice following the introduction of the *SMN*^*A111G*^ allele, which is capable of chaperoning snRNP assembly. Correction of SMA was dependent on snRNP assembly activity in the spinal cord (Workman et al., 2009). This study is in agreement with an earlier report demonstrating that motor neuron degeneration was rescued when purified snRNPs were injected in SMN-deficient zebrafish embryos (Winkler et al., 2005). Interestingly, in line with earlier findings demonstrating that knockdown of pICln or U1 snRNP leads to SMA-like defects in zebrafish (Winkler et al., 2005; Yu et al., 2015) (Table 1), disruption of either pICln or Tgs1 was found to result in motor defects that mirror those described on loss of SMN or Gemins in *Drosophila* (Borg et al., 2016). pICln and Tgs1 are two factors that are known to have a leading role in the early and late phase of snRNP assembly, respectively. Importantly, unlike the Gemins (reviewed in Cauchi, 2010), they have never been directly linked to the assembly and transport of messenger ribonucleoprotein (mRNP) complexes along axons, which is often considered as the primary non-canonical activity of the SMN-Gemins complex (reviewed in Donlin-Asp et al., 2016).

**Table 1:** Key studies in animal models linking motor dysfunction to perturbation in snRNP biogenesis.

**Organism**	**Genotype**	**Manipulation and/or findings**	**References**
*Drosophila*	Loss-of-function *Smn^*73Ao*^* mutants	Reduced snRNA levels; perturbation of the splicing and expression of genes with minor-class introns including *stasimon*	Lotti et al., 2012
*Drosophila*	Knockout of *Smn* (*Smn^*X7*^* mutants)	Synaptic dysfunction and muscle growth defects are rescued by transgenic expression of *stasimon*, a minor-class intron containing gene	Lotti et al., 2012
*Drosophila*	pICln or Tgs1 disruption via RNAi-mediated knockdown or overexpression	SMA-like motor system defects	Borg et al., 2016
Zebrafish	Antisense morpholino knockdown of pICln or U1 snRNP components U1-70K or U1 snRNA	SMA-like motor axon degeneration	Winkler et al., 2005; Yu et al., 2015
Zebrafish	Antisense morpholino knockdown of SMN	Injection of purified snRNPs prevents motor neuron degeneration; injection of the mRNA of genes that are misspliced in SMA including *Stasimon, Chondrolectin* or *Neurexin *2**, results in correction of SMA-like motor axon defects	Winkler et al., 2005; Lotti et al., 2012; See et al., 2014; Sleigh et al., 2014
Mouse	Knockout of mouse *Smn* and manipulation of Smn levels through introduction of human *SMN2, SMNΔ7* and/or *SMN^*A2G*^* transgenes	Degree of impaired snRNP assembly in spinal cord extracts is associated with disease severity; significant decrease in the levels of select snRNPs	Gabanella et al., 2007
Mouse	Knockout of mouse *Smn* and introduction of one or two copies of the human *SMN2* transgene	Introduction of the snRNP assembly competent human *SMN^*A111G*^* transgene rescued the disease phenotype; reduced expression and splicing of *Neurexin *2** in spinal cord; elevated retention of minor class introns that is corrected by a therapeutic ASO	Workman et al., 2009; See et al., 2014; Doktor et al., 2017
Mouse	Knockout of mouse *Smn* and introduction of the human *SMN2* and *SMNΔ7* transgenes	Symptomatic mice have tissue-specific alterations in snRNA levels and widespread pre-mRNA splicing defects in gene transcripts with diverse roles; altered splicing and reduced expression of *Stasimon*, a minor-class intron containing gene, in motor neurons and proprioceptive neurons of early-symptomatic mice; splicing abnormalities and expression-level changes of specific mRNAs critical for motor neuron function including synaptogenesis in laser-capture micro-dissected motor neurons of pre-symptomatic mice	Zhang et al., 2008, 2013; Baumer et al., 2009; Lotti et al., 2012
Mouse	Knockout of mouse *Smn*, introduction of the human *SMN2* transgene (4 copies) and intracerebroventricular administration of an ASO mediating the skipping of exon 7 from the human *SMN2* transgene	Extensive intron retention, particularly minor-class introns, in spinal cord extracts that was corrected by a therapeutic ASO; p53 activation; markers of DNA double-strand breaks in neurons of brain and spinal cord	Jangi et al., 2017

*SMN^Δ7^, predominant isoform produced by the human SMN2 gene*.

Consistent with a fundamental role for SMN in chaperoning snRNP assembly, several studies were successful in identifying splicing defects as a consequence of SMN loss and, importantly, explain how missplicing of specific transcripts leads to motor dysfunction in SMA. Whereas symptomatic SMA mice were shown to have widespread pre-mRNA splicing defects in numerous transcripts of diverse genes (Zhang et al., 2008; Baumer et al., 2009), at a pre-symptomatic stage, they exhibit dysregulation of genes that are critical for the function of the motor neuron and may thus contribute to SMA's signature pathology (Zhang et al., 2013). Similarly, in *Drosophila*, SMN deficiency perturbed the splicing and expression of several genes including that of *stasimon*, whose correct splicing is dependent on the minor spliceosome. Stasimon mRNA expression and splicing was also found perturbed in the constituent neurons of the sensory-motor circuit in SMA mice. Restoration of *stasimon* expression in the motor circuit was found to correct in part the motor system defects in *Drosophila Smn* mutants and *Smn*-deficient zebrafish, thus establishing stasimon as an SMN target gene (Lotti et al., 2012). Other studies have since focused on additional genes that are misspliced in SMA models including *Neurexin2* (See et al., 2014) and *Chondrolectin* (Sleigh et al., 2014), both of which are important for motor neuron axon outgrowth. Further still, following up on a previous study in a genetic model (Doktor et al., 2017), an ASO-inducible model of SMA was recently found to have widespread intron retention, particularly those spliced by the minor spliceosome, in spinal cord extracts. Importantly, these changes were rescued by a therapeutic ASO, thereby indicating that intron removal is directly correlated with SMN levels. Interestingly, intron retention was associated with a strong induction of the p53 pathway, and markers of DNA double-strand breaks were apparent in the neurons of the spinal cord and brain of SMA mice (Jangi et al., 2017). Thus, it is highly likely that instead of single gene effects, inefficiencies in pre-mRNA processing consequent to severe SMN deficiency lead to a DNA damage and stress response that compromises the survival of the motor neuron. Nonetheless, the reasons why the neuromuscular system remains highly vulnerable to damage warrants further investigation.

## Conclusion

The ample evidence linking defective chaperoning of snRNP assembly to neuromuscular dysfunction is not only consistent with SMA being a chaperonopathy but also sets the scene for the discovery of therapies that target this pathway. Inhibition of RNA decay pathways to correct snRNP levels (Shukla and Parker, 2014) or suppression of genome instability induced by intron retention (Jangi et al., 2017) are two treatment routes that are successful, at least *in vitro*. Although the effectiveness of these and other approaches on animal models or humans, remains to be investigated, the suppression of snRNP hypo-assembly and splicing defects may provide benefits to SMA patients beyond the benefit of SMN restoration alone. Considering that distrubances in snRNP assembly are also a component of the pathogenesis of the adult-onset amyotrophic lateral sclerosis (ALS) (Cauchi, 2014; Sun et al., 2015; Yu et al., 2015), such therapeutic strategies are expected to have broad implications in motor neuron disease.

## Author contributions

RC conceived the review focus; ML, NV, and RC conducted the literature review, wrote and edited the manuscript.

### Conflict of interest statement

The authors declare that the research was conducted in the absence of any commercial or financial relationships that could be construed as a potential conflict of interest.
